# Single- and Multiple-Dose Trials to Determine the Pharmacokinetics, Safety, Tolerability, and Sex Effect of Oral Ginsenoside Compound K in Healthy Chinese Volunteers

**DOI:** 10.3389/fphar.2017.00965

**Published:** 2018-01-11

**Authors:** Lulu Chen, Luping Zhou, Jie Huang, Yaqin Wang, Guoping Yang, Zhirong Tan, Yicheng Wang, Gan Zhou, Jianwei Liao, Dongsheng Ouyang

**Affiliations:** ^1^Department of Clinical Pharmacology, Xiangya Hospital, Central South University, Changsha, China; ^2^Institute of Clinical Pharmacology, Central South University, Changsha, China; ^3^Center of Clinical Pharmacology, Third Xiangya Hospital, Central South University, Changsha, China; ^4^Hunan Key Laboratory for Bioanalysis of Complex Matrix Samples, Changsha, China

**Keywords:** ginsenoside compound K, pharmacokinetics, rheumatoid arthritis, sex factor, tolerability

## Abstract

**Background and objectives:** Ginsenoside compound K (CK) is a candidate drug for rheumatoid arthritis therapy. The objective of this study was to investigate the pharmacokinetic properties, safety and tolerability of CK.

**Methods:** In randomized, double-blind trials, 76 healthy Chinese subjects received 1 of 7 single oral doses (25, 50, 100, 200, 400, 600, 800 mg) of CK or placebo under fasting condition, and another 36 subjects received repeated oral doses (100, 200, or 400 mg) of CK or placebo for up to 9 days a week after a corresponding single dose, after breakfast. Both sexes were equally represented in the two trials. Pharmacokinetic parameters of CK and its metabolite 20(S)-protopanaxadiol (PPD) were calculated and statistically analyzed according to the plasma concentration data. Tolerability was evaluated by adverse events (AEs) and laboratory examinations.

**Results:** The range of time to maximum concentration (T_max_) was 1.5–6.0 h, with a linear increase in the exposure of CK over the dose range of 100–400 mg. Steady state was reached after the 7th administration, and the accumulation index range was 2.60–2.78. Sex differences were characterized by a higher exposure in females than males with the single administration after breakfast. In addition, no severe AEs were observed.

**Conclusion:** CK was safe and well-tolerated over the treatment period. The sex- and food-related impacts on CK pharmacokinetics need further investigations to be validated. (Registration number: ChiCTR-TRC-14004824 and ChiCTR-IPR-15006107, http://www.chictr.org.cn/index.aspx).

## Introduction

**Figure 1:**
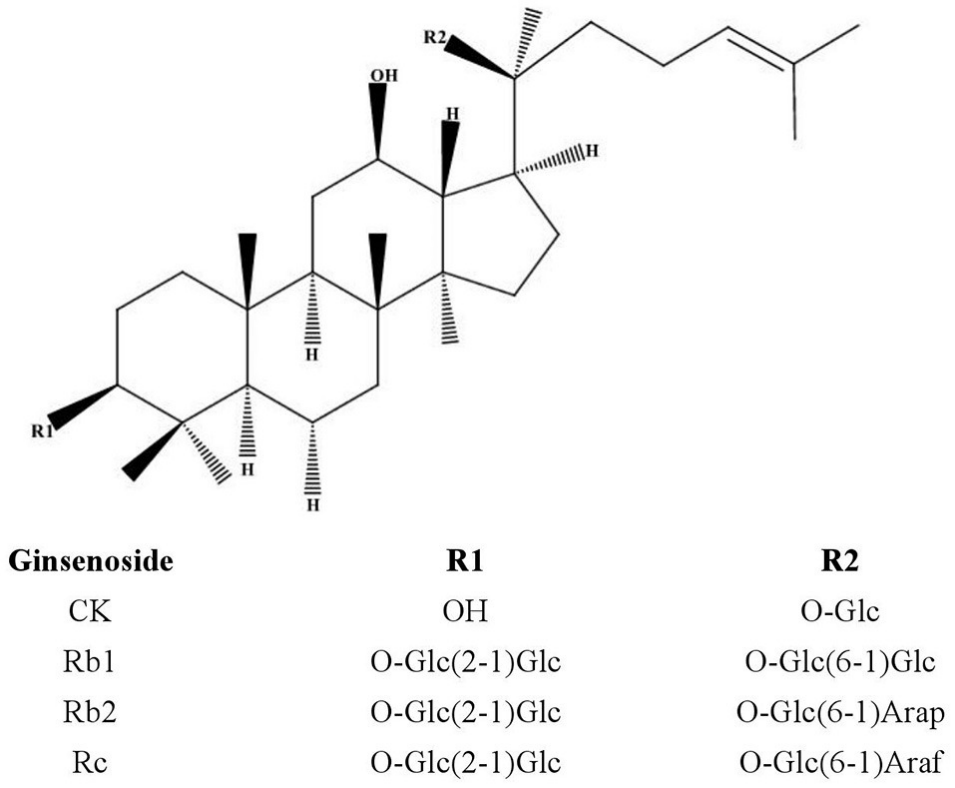
Chemical structures of protopanaxadiol-type ginsenosides. PPD, protopanaxadiol; Glc, D-glucopyranosyl; Arap, L-arabinopyranosyl; Araf, D-arabinofuranosyl.

Ginsenoside compound K (20-O-beta-D-glucopyranosyl-20(S)-protopanaxadiol, also known as M1, IH-901, and G-CK) belonging to the protopanaxadiol-type saponins (Figure 1) was first isolated by Japanese researchers in 1972 (Yosioka et al., 1972) and could be produced from ginsenosides, such as Rb1, Rb2, and Rc, through various conversion methods (Yang et al., 2015). While 20(S)-protopanaxadiol (PPD) is the hydrolysis end-product of deglycosylation reaction of natural PPD type ginsenosides. It has been reported that CK can be further disintegrated by gastric acid and/or intestinal microorganisms and enzyme conversion into 20(S)-PPD both *in vivo* and *vitro* (Tawab et al., 2003; Yoo et al., 2011; Oh and Kim, 2016). Researchers believed that the multiple pharmacological activities of ginsenosides are mediated mainly by their metabolic component, CK (Wakabayashi et al., 1997; Wang et al., 2012; Park et al., 2013). However, pure CK has not already been available as a drug in the world so far.

The anti-inflammatory activity of CK has been identified in several studies. Pre-clinical studies suggested that ginsenoside CK had a satisfactory anti-inflammatory effect through significant downregulation of the mRNA levels of tumor necrosis factor (TNF), interleukin 1 (IL1), interleukin 4 (IL4), interferon γ (INFG), and prostaglandin-endoperoxide synthase 2 (PTGS2) (Wang et al., 2016). Additionally, it has been reported that CK could clearly suppress the activation of mitogen-activated protein kinases (MAPKs) and Toll-like receptors 4 (TLR4)/lipopolysaccharide-induced nuclear factor κB and block pro-inflammatory cytokines secreted by macrophages (Wu et al., 2014). While Chen et al. observed the effect of CK on T-cell activation in mice with collagen-induced arthritis (Chen et al., 2015). The above results together with the considerable literature available (Cuong et al., 2009; Joh et al., 2011) suggest that CK may be a promising drug for the treatment of rheumatoid arthritis (RA). Therefore, Hisun Pharmaceutical Co., Ltd (Taizhou, China) aims to develop CK as an oral anti-RA drug after improving the preparation progress of pure CK (Yan et al., 2008). Currently, as a candidate drug for RA, Ginsenoside Compound K Tablet had been finished all phase 0 and phase I clinical trials under the approval (CDEL20130379) by the China Food and Drug Administration (CFDA).

Due to the fact that no CK preparation has been developed for human beings, ethical reasons lead to the pharmacokinetic (PK) study of CK in humans cannot be carried out. Although studies performed on healthy subjects after taking ginseng extract had been reported (Lee et al., 2009; Jin et al., 2012; Kim, 2013; Kim et al., 2013), knowledge of the absorption, distribution, metabolism and excretion (ADME) characteristics of pure CK is of evident interest. Meanwhile, previous clinical trials predominately used male subjects, thus ignoring the contribution of sex to the response measurements [Institute of Medicine (US) Forum on Neuroscience Nervous System Disorders, 2011; Institute of Medicine (US) Board on Population Health and Public Health Practice, 2012]. Potential sex-based PK differences remain a key consideration for all pharmacological agents. Dose adjustments or altered dosing schedules may be required in specific patient populations to maximize efficacy or minimize the risk of adverse effects if clinically relevant differences in the response to the drug exist. Moreover, from our previous studies performed on rats and a small number of healthy subjects, we found that CK displayed a potential sex dimorphism in pharmacokinetics after oral administration (Supplementary Figure 1 and Supplemental Table 1). With the increasing attention to sex and gender differences (USA, Report to Congressional Requesters, 2000; NIH, 2001; Bren, 2005; Clayton and Collins, 2014; Couzin-Frankel, 2014), we cannot ignore the phenomenon of sex differences in CK. Accordingly, another purpose of the present study is to investigate whether there is any difference in the pharmacokinetics of CK between healthy male and female subjects, and whether this kind of difference is consistent with what was previously observed.

This study was designed to determine the pharmacokinetics of CK and its metabolite 20(S)-PPD, as well as to investigate the safety, tolerability, and sex-related differences of CK after oral administration of CK at single and multiple daily rising doses.

## Methods

### Subjects

These two trials were approved with number 14,050 and 14,119 by the independent ethics committee of the Third Xiangya Hospital affiliated to Central South University. Subjects were evaluated for eligibility within 1 week before dosing. Evaluation included medical history, physical examination, clinical laboratory tests, vital signs, and ECG recording. Participants in these trials were healthy, non-smokers, and ranging in age from 18 to 45 years old, with a body mass index (BMI) of 19 to 24 kg·m^−2^ and minimum body weight of 45 and 50 kg for females and males, respectively. Additionally, eligible female subjects were required not to be pregnant, lactating, or of childbearing potential. Women who used contraceptive pills within 30 days and estrogen or progestin within 6 months were also excluded. Other exclusion criteria were as follows: (1) history of severe circulatory system, endocrine system, nervous system, digestive system, respiratory system, hematological, immunological, or psychiatric disease; (2) history of gastrointestinal conditions that could impact drug absorption and excretion; (3) recent exposure to over the counter, prescription, or investigational medication, especially any drugs that inhibit or induce drug metabolizing enzymes were prohibited (within 30 days); (4) drug or alcohol abuse (within 6 months); (5) subjects with positive tests for human immunodeficiency virus, hepatitis B virus surface antigen, or anti-hepatitis C virus antibody. Eligible subjects were apprised of the risks of the study and read, understood and signed the written informed consent forms prior to entry into the study and any study procedures.

All procedures performed in this study, involving human participants, were in accordance with the Good Clinical Practice Guidelines, as defined by the International Conference on Harmonization, the Declaration of Helsinki and its later amendments or comparable ethical standards.

### Drug and dosage justification

Ginsenoside Compound K Tablets (50 mg per tablet, lot CK1312004, expiration 2015-12-12) and placebo tablets (50 mg per tablet, lot FCK1312008, expiration 2015-12-12) were supplied by Hisun Pharmaceutical Co., Ltd (Taizhou, China). All drugs used throughout this study were kept in a secure, limited-access storage area under the recommended storage conditions until they were needed or until they were returned to the sponsor.

Dosage justification in single-dose pharmacokinetics: The starting dose in this study was defined according to the method for first-in-human clinical trials of new molecular entities in adult healthy volunteers recommended by the US Food and Drugs Administration (FDA). No observed adverse effect level (NOAEL) of CK in rats and beagle dogs is 25.0 and 7.5 mg·kg^−1^, respectively, and the human equivalent dose (HED) was calculated by normalization to body surface area. Finally, the maximum recommended starting dose (MRSD) was calculated with the safety factor defined as 10 and the body weight of human as 60 kg.

Diarrhea symptom occurred at the dose of 150 mg·kg^−1^ in Sprague Dawley (SD) rats and 75 mg·kg^−1^ in beagle dogs during the long-term toxicity test. The 1/5 dose was 30 and 15 mg·kg^−1^, respectively, following the maximum doses estimated from body weight were 1,800 and 900 mg. In summary, the maximum dose of this trial was ultimately set at 800 mg due to safety concerns.

Dosage justification in multiple-dose pharmacokinetics: CK was safe and well-tolerated at a single dose of 25, 50, 100, 200, 400, 600, 800 mg in healthy Chinese volunteers. Considering the linear range of CK exposure combined with the efficacy data of pharmacodynamic studies from our cooperative unit with the possible dose for clinical application (Wu et al., 2014), we finalized repeated oral doses of 100, 200, and 400 mg qd in this multiple-dose clinical trial. The placebo control was for assessment of safety/tolerability only.

### Study design

Both trials were double-blind, randomized, placebo-controlled studies conducted at the phase I unit (Center of Clinical Pharmacology, the Third Xiangya Hospital, Central South University, China).

Single-dose pharmacokinetics: The single-dose trial was performed first. Seven doses of CK were given orally under fasting condition: 25, 50, 100, 200, 400, 600, and 800 mg. Eligible subjects (*n* = 76, male:female = 1:1) were randomized into each dose level (*n* = 8, 8, 10, 10, 10, 8, 8, respectively) or placebo groups (*n* = 2 for each dose group) with equal number of men and women in each group. Subjects in each group were admitted to the clinical facility the day before drug administration and received the corresponding dose of the test drug or placebo with 250 ml warm water on the morning of the dosing day. Drinking before and after medication was prohibited for 2 h, but the amount and time of drinking were not strictly controlled at other times. All subjects had standard lunch and dinner (unified light diet) after medication 4 and 10 h.

Multiple-dose pharmacokinetics: The multiple-dose trial could be carried out on the condition that a single-dose of CK (25–800 mg) was safe and well tolerated in healthy Chinese volunteers. After breakfast, one of three doses (100, 200, or 400 mg) of CK or placebo were given to 36 eligible subjects randomized to each dose (male:female = 1:1), in which 10 were on active treatment and 2 were on matching placebo. CK were administered as a single dose on day 1 and then as repeated doses for 9 consecutive days (days 7–15 of the trial), and the drug was administered once-daily in the morning after meals.

Dose escalation was based on safety, tolerability from the previous dose and each subject only received either the test drug or placebo once in these two trials.

### Sampling

The sample collection points were designed according to the PK properties of ginsenoside CK according to pre-clinical data obtained from experiments performed on animals. In these clinical trials, blood samples (5 ml) for PK assessment were collected from an indwelling catheter or by direct venipuncture.

Single-dose pharmacokinetics: In the 25 and 50 mg dose groups, serial blood samples were collected at pre-dose (*t* = 0) and at 0.25, 0.5, 1, 1.5, 2, 2.5, 3, 3.5, 4, 5, 6, 8, 12, 24, 36, and 48 h post-dose. From the 100 to 800 mg dose groups, blood samples were additionally collected at 72 and 96 h post-dose. Plasma was separated from whole blood by refrigerated centrifugation (3,000 rpm, 10 min), within 1 h after sampling, transferred to labeled storage tubes, and stored at −70°C prior to workup and analysis. Urine samples were collected at different time periods: 0–2, 2–4, 4–6, 6–8, 8–10, 10–12, 12–24, and 24–48 h post-dose and the volume was measured immediately after collection from each subject. We preserved 8 ml specimens into 10 ml centrifuge tubes and stored at −70°C for PK assessment.

Multiple-dose pharmacokinetics: Blood samples were collected at pre-dose (*t* = 0) and at 0.25, 0.5, 1, 1.5, 2, 2.5, 3, 3.5, 4, 5, 6, 8, 12, 24, 36, 48, 72, and 96 h post-dose on day 1 and day 15. Other blood samples were collected before drug administrations on the morning of day 13 and day 14, for the determination of minimum plasma concentrations at steady state (C_min,ss_).

### Bioanalytical assay

All samples were preserved at −70°C and analyzed with a LC-MS/MS (API 4000 LC-MS/MS, ABI Company, Foster City, CA, USA) at the Institute of Clinical Pharmacology, Central South University (Changsha, China). Concentrations of CK (Hisun Pharmaceutical Co., Ltd, Taizhou, China) and 20(S)-PPD (National Institutes for Food and Drug Control, Beijing, China) were measured using a triple quadrupole tandem mass spectrometer in multiple reaction monitoring (MRM) mode using a positive electrospray ionization source at m/z 621.4→ 160.8 for the CK and m/z 461.5→ 425.3 for 20(S)-PPD with a dwell time of 200 ms.

An aqueous solution of a stable internal standard (IS for CK: digoxin, Shijiaoke Biological Technology Co. Ltd., Beijing, China; IS for PPD: coumarin, Sigma-Aldrich, St. Louis, USA) and 1 M phosphate buffer were added to 0.5 ml of thawed sample, and analytical standards and quality control (QC) samples were prepared in the same matrix. All samples were treated with 2 ml of methyl tert-butyl ether (MTBE, CNW technologies GmbH, Duesseldorf, Germany) to precipitate the bulk of plasma proteins. After mechanical shock by mixing for 10 min and a subsequent centrifugation (4,000 rpm, 4°C, 10 min), 1.4 ml of the supernatant was transferred and blow-dried with nitrogen in a water bath at 40°C. Lastly, the residue was dissolved in 100 μl of mobile phase and then was subjected to a mechanical shock by mixing, and centrifuged. The supernatant was subsequently injected into the column for analysis. The CK, 20(S)-PPD and the ISs were separated from the matrix components using a HyPURITY C18 (150 × 2.1 mm, 5 μm, Thermo Hypersil-Keystone, Bellefonte, PA, USA) with mobile phase composed of acetonitrile and aqueous ammonium acetate. The lower limits of quantification (LLOQ) were 1.00 ng·ml^−1^ (CK), 0.15 ng·ml^−1^ (PPD), and 2.79 ng·ml^−1^ (CK), 0.15 ng·ml^−1^ (PPD), in the single-dose trial and multiple-dose trial, respectively. Accuracy and precision of the assay were determined by evaluating the performance of the assay controls. Over the quantification range, the accuracy of the QC samples varied from 85 to 115% and the intra- and inter-assay imprecision was <15%.

The processing method of the urine samples was similar to the method used for the plasma samples. For CK, the only difference was in the mobile phase (acetonitrile: 10 mM aqueous ammonium acetate = 50:50, V/V, for plasma samples; acetonitrile: 20 mM aqueous ammonium acetate = 60:40, V/V, for urine samples; acetonitrile, Merck KGaA, Darmstadt, Germany; ammonium acetate, CNW technologies GmbH, Duesseldorf, Germany). As for 20(S)-PPD, neither the IS nor the mobile phase were the same (IS: coumarin for plasma samples and finasteride for urine samples; mobile phase was acetonitrile: 10 mM aqueous ammonium acetate = 50: 50, V/V, for plasma samples, and acetonitrile: 0.1% formic acid aqueous solution = 50: 50, V/V, for urine samples). The calibration curve range for CK was linear from 1.00 to 1002.0 ng·ml^−1^ with a correlation coefficient equal to 0.9984, and from 0.15 to 54.30 ng·ml^−1^ with a correlation coefficient equal to 0.9978 for 20(S)-PPD. Three levels of QC samples (2.00, 50.10, or 801.60 ng.ml^−1^ CK) were prepared in urine for testing accuracy and coefficient of variations (CVs), and the values were 94–103% and 1.06–6.54%, respectively.

All validation results showed that the above-described methods can be used to determine plasma and urine concentrations of ginsenoside CK and 20(S)-PPD.

### Safety assessments

Safety was evaluated by continuous observation of adverse events (AEs), monitoring of vital signs, ECG, hematology, biochemistry, and urinalysis at baseline, during the trials at the set time points following drug administration, and at follow-up visits after study completion. AEs were classified by their intensity into mild, moderate and severe. Additionally, the AEs were also classified according their relationship to the test drug as positive, probable, possible, remote, or unrelated. The severity of the AEs, as well as their relationship to test drug, was assessed by a qualified physician in the unit. Clinically relevant changes in vital signs, ECG and laboratory parameters were defined as marked abnormalities. ECG were recorded in the semisupine position after a rest of at least 5 min. It is worth noting that all the information, including symptoms and signs after dosing or the medications that the subjects were concomitantly taking must be recorded on the case-report form by investigators. All available data from subjects who received the test drug and placebo were included in the summaries of the safety data and were descriptively analyzed. In addition, the differences between laboratory parameters at baseline and after drug treatment were analyzed to determine whether there were clinical significances.

### Pharmacokinetic analyses

PK parameters were derived according to noncompartmental analysis and actual elapsed time from dosing by WinNonlin version 6.1 (Pharsight Corporation, Mountain View, CA, USA).

Experimental observations provided the plasma CK and 20(S)-PPD concentrations vs. the time data after single-dose administration, including maximum plasma concentration (C_max_) and the time to maximum plasm concentration (T_max_). The area under the curve (AUC) from time zero to the last time point (AUC_last_) and the AUC extrapolated to infinity (AUC_inf_) were calculated using the linear trapezoidal rule. The slope (elimination rate constant, K) of the terminal phase of the plasma concentration-time profile was determined, using a weighting factor of 1, by the method of least squares (log-linear regression of at least three data points). The t_1/2_ was estimated as (ln2)/K. The apparent plasma clearance of the drug after oral administration (CL/F) was expressed as a function of bioavailability (F) and calculated as the dose divided by AUC_inf_, assuming that the complete systemic bioavailability F = 1. The apparent volume of distribution (V/F) was equal to (CL/F)/K. The mean residence time (MRT) was calculated as the area under the first moments curve (AUMC) divided by AUC. The dose-normalized PK parameters e.g., C_max_/D and AUC_last_/D, were obtained by dividing each PK result by the administered dose. The cumulative excretion rate was calculated as the amount of unchanged drug in the urine over a time interval divided by the dose and was expressed as thousand points (‰).

On day 15 in the multiple-dose trial, the PK parameters, such as t_1/2_, CL/F, and V/F at steady state were calculated using the same method as that used for the single-dose trial. In addition, the T_max_ at steady state (T_max,ss_), C_max_ at steady state (C_max,ss_), and C_min,ss_ were obtained from experimental observation after the steady state during the multiple-dose trial. The AUC for dosing interval was expressed as AUC_τ_, where τ is the dosing interval (24 h). The average steady-state drug concentration (C_avg_) is calculated as AUC_τ_/τ, and the fluctuation ratio was calculated as (C_max,ss_ − C_min,ss_)/C_avg_. The accumulation index was calculated using dosing interval and elimination rate constant as in the following equation: 1/(1 − e^−Kτ^).

Above these PK parameters were calculated for each subject.

### Statistical analyses

All PK data were summarized using descriptive statistics. Statistical analyses were performed using the SPSS software version 22.0 (SPSS Inc., Chicago, IL, USA). Values are expressed as the mean ± *SD* for all parameters with the exception of T_max_, which is presented as the median (range).

Dose proportionality was explored on logarithmic transformed C_max_ and AUC parameters using the power model, PK = A × (dose)^β^, where PK is the pharmacokinetic parameter, A is the intercept and β is the dose-proportionality coefficient. The 95% confidence interval (CI) of β was then calculated. Note that a slope of 1 would correspond to perfect dose proportionality. Possible deviations from dose proportionality in the exposure were also assessed by ANOVA applied on the logarithmic transformed C_max_/D, AUC_last_/D, AUC_inf_/D, and AUC_τ_/D. In the second trial, the natural logarithm of the pre-dose concentrations on days 13, 14, and 15 were analyzed to determine the achievement of a steady state. The Kruskal–Wallis *H*-Test was used to establish whether significant differences in pre-dose concentrations existed among the three target dose days, and regression analysis was used to evaluate whether the slope of the pre-dose concentrations vs. days curve was significantly different from zero in the three dose groups.

PK parameters (Logarithmic transformed PK parameters) were compared between day 1 and day 15 by ANOVA to assess the effect of repeated administration of CK, as well as the sex-related impact. The 90% CIs for the ratios of the geometric means of the exposure parameters were obtained by back transformation. Simultaneously, a non-parametric test was performed on the T_max_ and cumulative excretion rate (‰) of ginsenoside CK. In addition, a two-way ANOVA combined with interaction plots was performed to examine the interaction effect of repeated administration and sex on the pharmacokinetics of CK.

All the safety data, including vital signs, laboratory indexes and ECG, were descriptively analyzed, and the statistical analyses were conducted to evaluate the effects of the test drug by comparing the differences between before and after drug treatment, between the test drug group and the placebo group and among the different dose groups.

An alpha of 0.05 was used for any hypothesis test performed.

## Results

### Demographic characteristics

A total of 112 healthy volunteers, half of whom were male and half of whom were female, participated in these trials (76 in the single-dose trial and 36 in the multiple-dose trial) after each one of them provided written informed consent (one subject in each of the two trials withdrew their informed consent before taking the drug, thus, we enrolled two eligible candidate subjects immediately). All the participants received one dose of CK or placebo, and completed the PK assessments. The demographic characteristics at baseline of the subject population in the two trials are summarized in Table 1.

**Table 1:** Demographic characteristics of subjects.

	**Single-dose trial**								**Multiple-dose trial**			
	**25 mg (*n* = 8)**	**50 mg (*n* = 8)**	**100 mg (*n* = 10)**	**200 mg (*n* = 10)**	**400 mg (*n* = 10)**	**600 mg (*n* = 8)**	**800 mg (*n* = 8)**	**Placebo (*n* = 14)**	**100 mg (*n* = 10)**	**200 mg (*n* = 10)**	**400 mg (*n* = 10)**	**Placebo (*n* = 6)**
**AGE (years)**												
Mean ± (*SD*)	24 ± 2	23 ± 3	21 ± 2	21 ± 3	22 ± 3	23 ± 3	23 ± 3	22 ± 3	24 ± 3	24 ± 4	23 ± 2	24 ± 3
Range	22–27	19–27	18–24	18–26	18–26	19–26	19–27	19–28	18–27	19–28	20–27	20–27
**BODY WEIGHT (kg)**												
Mean ± (SD)	56.1 ± 8.0	57.4 ± 7.5	53.3 ± 6.8	58.9 ± 9.7	57.0 ± 9.6	56.1 ± 6.3	54.7 ± 6.1	57.0 ± 7.6	61.3 ± 6.9	54.3 ± 6.3	54.0 ± 7.2	58.9 ± 7.5
Range	45–68	51–70	47–70	47–77	48–75	48–63	48–65	45–69	53–74	46–68	45–64	52–70
**HEIGHT (m)**												
Mean ± (*SD*)	1.63 ± 0.09	1.66 ± 0.05	1.61 ± 0.06	1.66 ± 0.08	1.64 ± 0.10	1.62 ± 0.07	1.62 ± 0.07	1.64 ± 0.08	1.67 ± 0.08	1.62 ± 0.10	1.61 ± 0.09	1.65 ± 0.09
Range	1.49–1.75	1.59–1.73	1.55–1.74	1.54–1.80	1.51–1.80	1.53–1.70	1.52–1.71	1.52–1.78	1.56–1.77	1.48–1.78	1.47–1.77	1.56–1.76
**BMI (kg**·**m**^−2^**)**												
Mean ± (*SD*)	21.0 ± 1.5	20.9 ± 2.0	20.4 ± 1.3	21.3 ± 2.0	21.0 ± 1.7	21.3 ± 1.3	20.8 ± 1.5	21.1 ± 1.4	22.0 ± 0.9	20.7 ± 1.1	20.7 ± 1.5	21.5 ± 1.2
Range	19.0–23.5	19.0–23.9	19.2–23.1	19.1–23.8	19.1–23.9	19.3–23.1	19.2–23.6	19.0–23.3	20.2–23.9	19.2–22.4	19.1–24	19.7–23.1

*The ratio of male to female was 1:1 in each dose group. BMI, body mass index. Values are presented as mean ± SD and range. SD, standard deviation*.

One-way ANOVA was performed on the demographic data of the subjects to compare the differences among the dose groups in each trial, between the test drug group and the placebo group, and between the sexes (data not presented). The results revealed that there were no statistically differences in age, height, weight or BMI among the different dose groups, as well as between the test drug and the placebo groups (*p* > 0.05). It is worth mentioning that the height and weight were significantly different (*p* < 0.05), between male and female subjects, but not the BMI.

### Pharmacokinetic parameters

#### Single-dose trial

The PK properties of CK and 20(S)-PPD were studied after single oral administration of CK at doses ranging from 25 to 800 mg. Based on the pooled data above from the 62 subjects who took the test drug and completed all sampling, which were included in the PK analyses, we observed a surprising phenomenon that the average C_max_, AUC_last_, and AUC_inf_ in the 600 mg group were larger than in the 800 mg group. Accordingly, we examined the extreme values by Boxplot in the SPSS software. The results suggested that a female subject should be excluded from the 25, 100, and 600 mg dose group.

**Figure 2:**
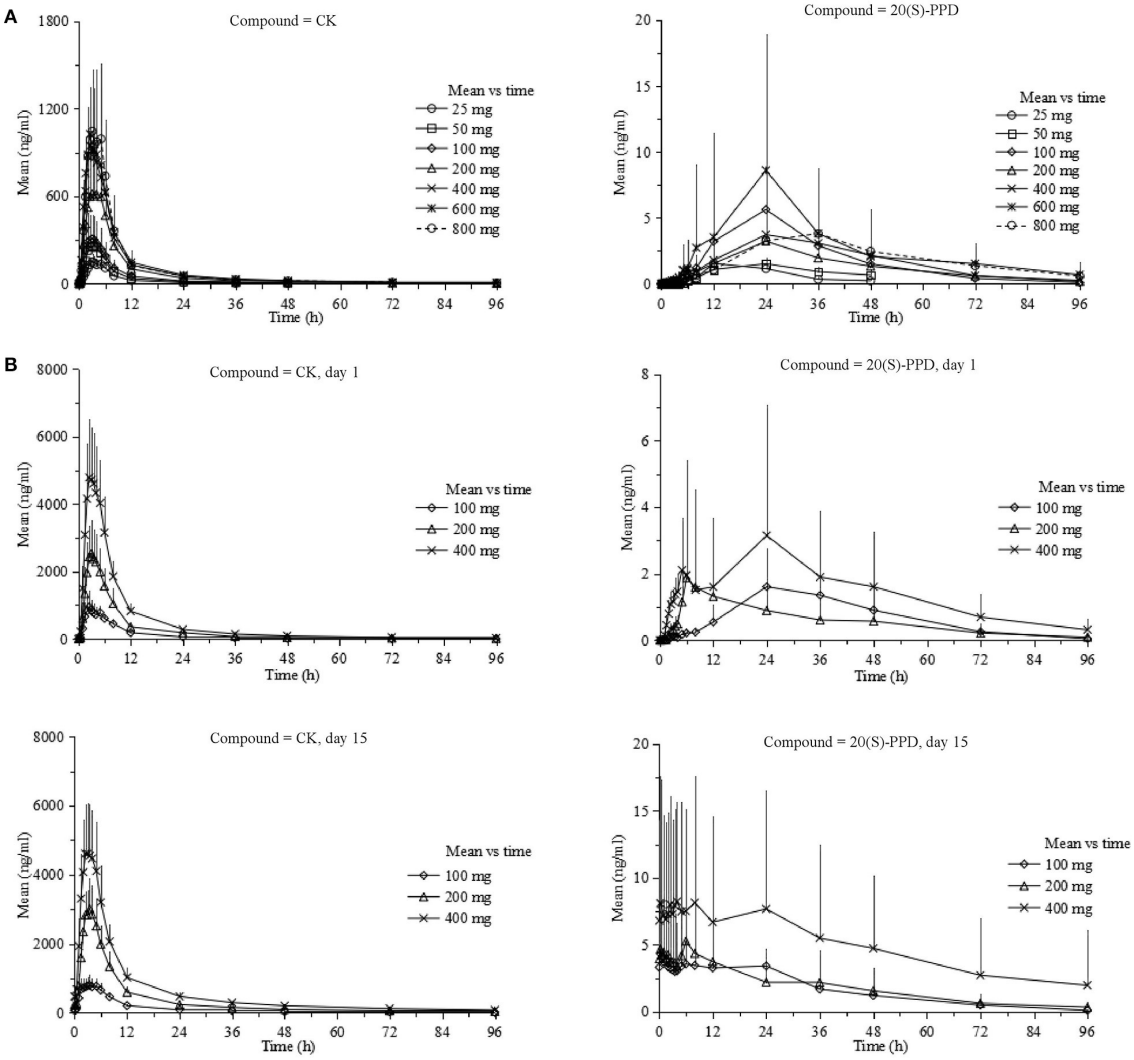
Pharmacokinetic profiles (Mean ± *SD*) of CK and 20(S)-PPD in single-dose trial **(A)** and multiple-dose trial **(B)**, respectively. CK, compound K; PPD, protopanoxadiol.

**Figure 3:**
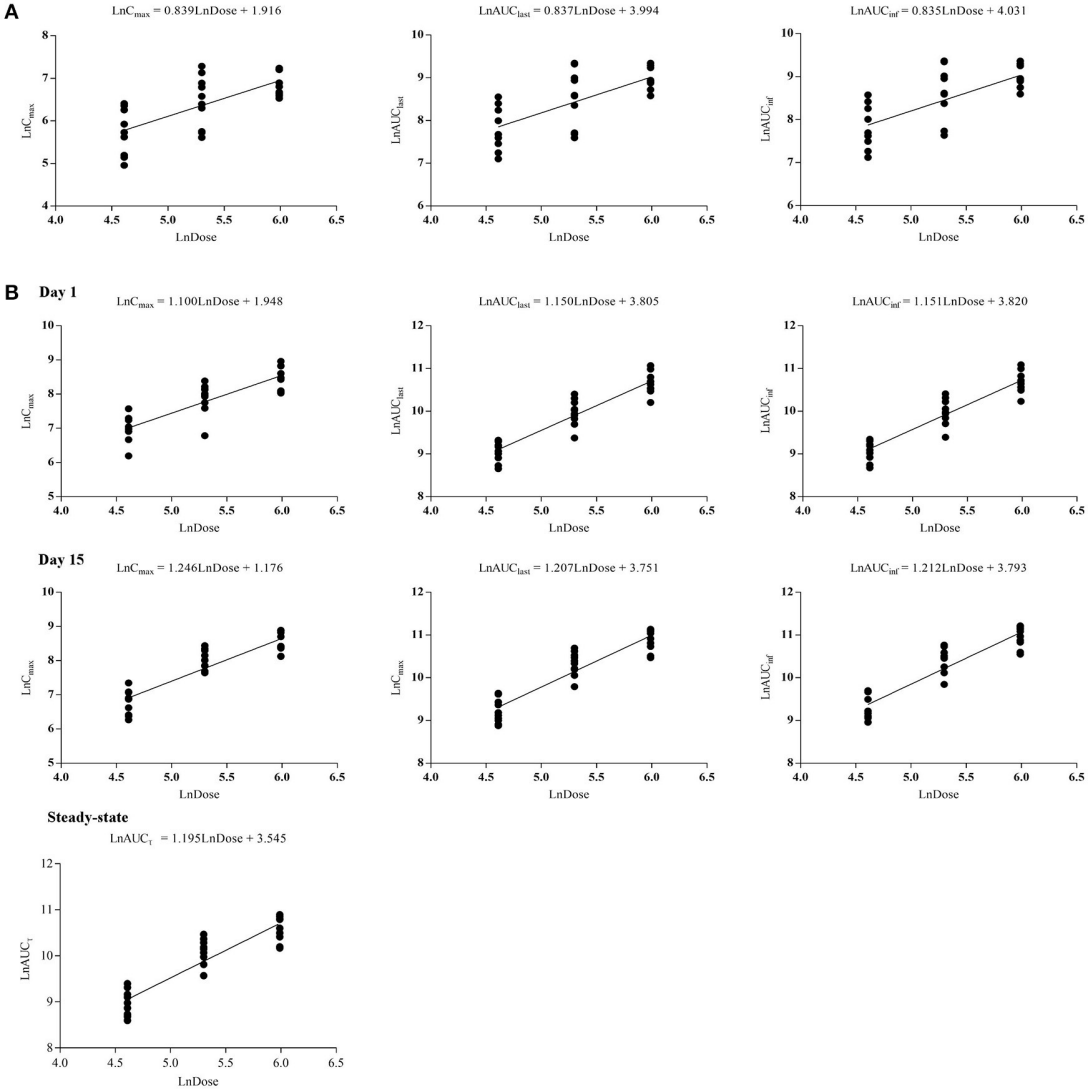
Dose proportionalities of C_max_, AUC_last_, AUC_inf_, and AUC_τ_ of CK in single-dose trial **(A)** and multiple-dose trial **(B)**, respectively. CK, compound K.

The curves of the mean plasma concentration-time of CK and its metabolite 20(S)-PPD in the single-dose trial are presented in (Figure 2A). The PK parameters of CK and 20(S)-PPD after the single rising dose administration are summarized in Table 2. Clinical pharmacokinetics of CK were characterized by a median T_max_ varying from 2.5 to 3.3 h across the dose groups, the mean C_max_ values ranging from 175.1 ng·ml^−1^ (25 mg) to 1183.2 ng·ml^−1^ (800 mg), the mean AUC_last_ values ranging from 1225.9 h·ng·ml^−1^ (25 mg) to 9093.6 h·ng·ml^−1^ (600 mg), and the mean t_1/2_ ranging from 13.5 to 26.2 h. In comparison, the exposure to 20(S)-PPD was much lower than CK, with mean C_max_ and AUC_last_ values ranging from 1.6 ng·ml^−1^ (25 mg) to 9.5 ng·ml^−1^ (600 mg), and 32.4 h·ng·ml^−1^ (25 mg) to 269.8 h·ng·ml^−1^ (600 mg), respectively. The ratio of the total amount of CK in the urine to the dose was 0.10‰ (600 and 800 mg) to 0.46‰ (50 mg). The evaluation of the dose proportionality, was obtained from the slope β from the power model, is given in Table 3. Over the dose range of 25–800 mg, the 95% CI of the dose proportionality constant β for C_max_, AUC_last_, and AUC_inf_ of CK were not between 0.8 and 1.25. However, over the dose range of 100–400 mg, the values of β were between 0.8 and 1.25, suggesting that C_max_, AUC_last_ and AUC_inf_ of CK linearly increased from 100 to 400 mg. In addition, the dose-normalized C_max_, AUC_last_, and AUC_inf_ of CK were not significantly different among the 100, 200, and 400 mg groups. The graphs of all the dose linear correlation of CK are shown in Figure 3A.

**Table 2:** Pharmacokinetic parameters of CK and 20(S)-PPD.

**Parameters**	**Dose groups in single-dose trial**							**Dose groups in multiple-dose trial**					
	**25 mg**	**50 mg**	**100 mg**	**200 mg**	**400 mg**	**600 mg**	**800 mg**	**100 mg (*****n*** = **10)**		**200 mg (*****n*** = **10)**		**400 mg (*****n*** = **10)**	
	**(*n* = 7^a^)**	**(*n* = 8)**	**(*n* = 9^a^)**	**(*n* = 10)**	**(*n* = 10)**	**(*n* = 7^a^)**	**(*n* = 8)**	**Day 1**	**Day 15**	**Day 1**	**Day 15**	**Day 1**	**Day 15**
**CK**													
C_min_ (ng·ml^−1^)	–	–	–	–	–	–	–	–	333.4 ± 96.7	–	1029.7 ± 261.9	–	1725.4 ± 431.9
C_avg_ (ng·ml^−1^)	–	–	–	–	–	–	–	–	61.5 ± 13.0	–	203.1 ± 44.7	–	435.1 ± 112.2
C_max_ (ng·ml^−1^)	175.1 ± 35.1	300.4 ± 98.5	351.0 ± 180.0	733.9 ± 408.4	1031.0 ± 295.1	1157.5 ± 466.4	1183.2 ± 445.1	1116.3 ± 410.7	934.3 ± 322.9	2885.0 ± 983.8	3177.3 ± 877.0	5055.8 ± 1654.6	5165.7 ± 1498.1
T_max_ (h)	3.0 (1.5–5.0)	3.0 (2.0–4.0)	3.0 (2.0–6.0)	3.3 (2.5–5.0)	3.0 (2.0–6.0)	2.5 (1.5–5.0)	2.5 (1.5–5.0)	2.5 (1.5–6.0)	3.8 (1.5–5.0)	3.0 (2.0–5.0)	3.5 (2.5–5.0)	3.0 (2.0–4.0)	2.5 (1.5–5.0)
AUC_last_ (h·ng·ml^−1^)	1225.9 ± 266.9	2117.6 ± 770.3	2762.7 ± 1416.7	5960.8 ± 3524.4	8097.8 ± 2024.7	9093.6 ± 4200.3	8992.2 ± 4098.3	8876.8 ± 1990.0	10447.9 ± 3008.1	22252.8 ± 6322.0	31644.8 ± 8125.8	43889.3 ± 11091.7	55229.6 ± 12838.5
AUC_inf_ (h·ng·ml^−1^)	1271.2 ± 276.0	2249.5 ± 861.0	2821.0 ± 1440.4	6094.2 ± 3598.4	8243.9 ± 2050.2	9275.3 ± 4237.8	9199.5 ± 4178.2	9069.9 ± 2060.5	11144.3 ± 3229.3	22671.1 ± 6348.7	33995.8 ± 9063.6	44864.9 ± 11212.2	59151.1 ± 13313.7
AUC_τ_ (h·ng·ml^−1^)	–	–	–	–	–	–	–	–	8000.9 ± 2319.9	–	24711.6 ± 6285.2	–	41408.8 ± 10366.4
AUMC_τ_ (h·ng·ml^−1^)	–	–	–	–	–	–	–	–	60141.5 ± 17696.2	–	179177.1 ± 49122.9	–	306621.5 ± 78711.0
t_1/2_ (h)	13.5 ± 0.6	15.9 ± 4.2	22.4 ± 3.3	21.6 ± 5.5	22.3 ± 4.0	26.2 ± 6.3	25.7 ± 3.0	25.2 ± 5.6	34.6 ± 10.5	23.4 ± 7.9	37.3 ± 12.3	26.1 ± 3.3	34.2 ± 5.3
V/F (l)	397 ± 86	559 ± 185	1381 ± 546	1287 ± 563	1613 ± 379	3057 ± 2094	3993 ± 2136	402 ± 54	661 ± 242	326 ± 137	455 ± 152	358 ± 118	514 ± 188
CL/F (l·h ^−1^)	20.5 ± 4.7	25.9 ± 11.2	44.5 ± 21.6	47.7 ± 31.2	51.2 ± 12.3	76.1 ± 32.1	106 ± 50.3	11.6 ± 3.0	13.4 ± 3.6	9.6 ± 3.1	8.6 ± 2.5	9.4 ± 2.4	10.3 ± 3.0
MRT_last_ (h)	8.9 ± 1.3	9.1 ± 0.7	11.4 ± 0.9	11.5 ± 1.4	11.9 ± 1.1	12.7 ± 0.9	12.0 ± 1.8	12.2 ± 1.4	–	12.6 ± 1.6	–	12.5 ± 1.0	–
MRT_inf_ (h)	10.9 ± 1.9	12.5 ± 2.4	13.7 ± 1.0	13.8 ± 1.6	14.0 ± 1.5	15.9 ± 2.8	14.7 ± 2.6	14.6 ± 2.1	17.0 ± 2.2	14.9 ± 2.0	16.1 ± 1.6	15.2 ± 1.5	17.9 ± 1.8
Ae (‰)	0.41 ± 0.29	0.46 ± 0.34	0.25 ± 0.13	0.27 ± 0.15	0.19 ± 0.13	0.10 ± 0.08	0.10 ± 0.04	–	–	–	–	–	–
Fluctuation (%)	–	–	–	–	–	–	–	–	259.42 ± 48.85	–	289.25 ± 35.75	–	273.98 ± 37.16
Accumulation index	–	–	–	–	–	–	–	2.62 ± 0.62		2.78 ± 0.73		2.60 ± 0.31	
**20(S)-PPD**													
C_max_ (ng·ml^−1^)	1.6 ± 1.2	1.8 ± 0.6	6.3 ± 4.2	3.5 ± 2.7	4.8 ± 4.3	9.5 ± 9.8	5.1 ± 5.3	1.8 ± 1.1	5.0 ± 2.5	2.3 ± 3.4	5.7 ± 6.1	4.3 ± 3.6	11.0 ± 10.2
T_max_ (h)	12.0 (8.0–24.0)	24.0 (12.0–24.0)	24.0 (12.0–36.0)	24.0 (6.0–36.0)	24.0 (5.0–48.0)	36.0 (24.0–72.0)	30.0 (3.0–72.0)	24.0 (24.0–36.0)	5.5 (0.3–24.0)	7.0 (4.0-48.0)	6.0 (0.3–24.0)	16.0 (3.0–36.0)	5.5 (0.5–8.0)
AUC_last_ (h·ng·ml^−1^)	32.4 ± 26.2	43.9 ± 12.0	167.6 ± 125.0	117.8 ± 94.9	157.1 ± 174.6	269.8 ± 251.4	179.1 ± 171.0	60.6 ± 44.9	154.5 ± 81.8	54.1 ± 58.8	170.9 ± 191.0	133.1 ± 127.6	463.1 ± 512.3

*C_min_, minimum concentrations; C_avg_, average concentration; C_max_, maximum plasma concentration; T_max_, time to maximum plasma concentration; AUC_last_, area under the plasma concentration-time curve from zero to the time of the last quantifiable concentration; AUC_inf_, area under plasma concentration-time curve from zero to infinity; AUCτ, area under the plasma concentration-time curve from zero to 24 h; AUMCτ, area under the first moment plasma concentration-time curve from zero to 24 h; t_1/2_, terminal half-life; V/F, apparent volume of distribution after extravascular administration; CL/F, the apparent plasma clearance of the drug after extravascular administration oral; MRT_last_, mean residence time from time zero to the time for the last measurable concentration; MRT_inf_, mean residence time from time zero to infinity; Ae (‰), accumulative urine excretion rate*.

a *One subject was excluded. All values are presented as mean ± SD, except for T_max_, which is expressed as median (range). SD, standard deviation; CK, compound K; PPD, protopanoxadiol*.

**Table 3:** Dose proportionalities of CK and 20(S)-PPD.

**Parameters**	**Slope** ***β***^a^ **(95% CI)**	
	**CK**	**20(S)-PPD**
**SINGLE-DOSE TRIAL**		
**25**–**800 mg**		
C_max_	0.574 (0.476~0.671)	0.278 (0.055~0.501)
AUC_last_	0.599 (0.495~0.703)	0.420 (0.147~0.693)
AUC_inf_	0.593 (0.489~0.697)	0.392 (0.091~0.692)
**100**–**400 mg**		
C_max_	0.839 (0.506~1.172)	−0.186 (−0.810~0.438)
AUC_last_	0.837 (0.498~1.177)	−0.114 (−0.856~0.629)
AUC_inf_	0.835 (0.497~1.172)	**–**0.129 (−0.970~0.712)
**MULTIPLE-DOSE TRIAL**		
**Day 1**		
C_max_	1.100 (0.838~1.361)	0.522 (0.006~1.039)
AUC_last_	1.150 (0.975~1.326)	0.478 (−0.172~1.129)
AUC_inf_	1.151 (0.976~1.327)	0.352 (−0.347~1.050)
**Day 15**		
C_max_	1.246 (1.011~1.480)	0.313 (−0.270~0.896)
AUC_last_	1.207 (1.011~1.403)	0.490 (−0.127~1.106)
AUC_inf_	1.212 (1.015~1.409)	0.649 (0.026~1.271)

*C_max_, maximum observed concentration; AUC_last_, area under the plasma concentration-time curve from zero to the time of the last quantifiable concentration; AUC_inf_, area under plasma concentration-time curve from zero to infinity; CI, confidence interval; CK, compound K; PPD, protopanoxadiol*.

a *Power model equation: PK = A × dose^β^. β value between 0.8 and 1.25 indicates linearity*.

#### Multiple-dose trial

The PK properties of CK and 20(S)-PPD were studied after repeated oral doses of CK (100, 200, or 400 mg) qd for up to 9 days after a week of the corresponding single dose of CK both under the same dietary condition. A total of 30 (male:female = 1:1) subjects who were administered test drugs were included in the pharmacokinetics analysis. The mean plasma concentration vs. time curves of CK and 20(S)-PPD in this multiple-dose trial are shown in Figure 2B.

The PK parameters of CK and 20(S)-PPD, following a single oral administration of 100, 200, or 400 mg CK on day 1, are summarized in Table 2. CK was absorbed with a dose-independent median T_max_ ranging from 2.5 to 3.0 h, and the mean t_1/2_ varying from 23.4 to 26.1 h. Both the C_max_ and AUC of CK increased with rising dose up to 400 mg of CK. C_max_ ranged from 1116.3 ± 410.7 ng·ml^−1^ (100 mg) to 5055.8 ± 1654.6 ng·ml^−1^ (400 mg), and AUC_last_ ranged from 8876.8 ± 1990.0 (100 mg) to 43889.3 ± 11091.7 h·ng·ml^−1^ (400 mg). The mean C_max_ and AUC_last_ of the metabolite 20(S)-PPD ranged from 1.8 ng·ml^−1^ (100 mg) to 4.3 ng·ml^−1^ (400 mg), and 54.1 h·ng·ml^−1^ (200 mg) to 133.1 h·ng·ml^−1^ (400 mg), respectively. Thus, the concentration of metabolite was only about 0.08–0.16% of the parent drug. Moreover, a dose dependent increase in C_max_, AUC_last_, and AUC_inf_ of CK over the entire sampling period was indicated by the slope β ranging from 0.8 to 1.25 from the power model (Table 3).

After multiple doses of CK, specifically 100, 200, or 400 mg over 9 days, the PK parameters of CK and 20(S)-PPD were derived by noncompartmental analysis and are listed in Table 2. The Kruskal–Wallis *H*-test results demonstrated that no significant differences in the pre-dose concentrations of CK (*p* > 0.05) existed between day 13 and day 14 in the three dose groups. Regression analysis of logarithmic transformed pre-dose concentrations showed that the slope of the pre-dose concentrations vs. day curves was not significantly different from zero (*p* > 0.05), which also indicated that a steady state was reached. At steady state, AUC_τ_ exhibited a linear increase with dose.

After multiple doses of CK over 9 days, the exposure to CK proportionally increased with the rising dose (Table 3), and no statistically significant evidence was detected against dose proportionality of the pharmacokinetics across the 100–400 mg dose (*p* > 0.05). The dose linear correlation graphs of CK are presented in Figure 3B. The PK parameters of CK on day 15 were compared with those on day 1 to evaluate the impact of repeated administrations on the pharmacokinetics. Except for C_max_/D, and T_max_, the following PK parameters of CK on day 15 were significantly higher than those on day 1, including the dose-normalized AUC_last_ (133.59 ± 40.26 vs. 103.25 ± 27.94 h·ng·ml^−1^·mg^−1^), dose-normalized AUC_inf_ (143.10 ± 43.67 vs. 105.41 ± 28.29 h·ng·ml^−1^·mg^−1^), and t_1/2_ (35.3 ± 9.6 vs. 24.9 ± 5.8 h) (Table 4). Additionally, after 9 consecutive days receiving the 100, 200, or 400 mg dose, the daily fluctuations in plasma concentration were 259.42, 289.25, and 273.98%, and the mean accumulation indexes were 2.62, 2.78, and 2.60, respectively. This indicated that CK had a moderate accumulation across doses under the used regimen.

**Table 4:** Pharmacokinetic parameters of CK according to the time of administration or sex in the multiple-dose trial.

**Parameters**	**Day 1**	**Day 15**	**GMR (95% CI)**	**Day 1**			**Day 15**		
				**Male**	**Female**	**GMR (95% CI)**	**Male**	**Female**	**GMR (95% CI)**
C_max_/D	12.74 ± 4.46	12.71 ± 4.58	1.00 (0.82–1.22)	11.00 ± 4.25	14.49 ± 4.07^*^	1.37 (1.04–1.80)	11.18 ± 5.19	14.25 ± 3.38^*^	1.36 (1.04–1.76)
T_max_ (h)	3.0 (1.5 ± 6.0)	3.5 (1.5 ± 5.0)		3.0 (1.5–5.0)	3.0 (1.5–6.0)		3.5 (1.5–5.0)	3.5 (1.5–5.0)	
AUC_last_/D	103.25 ± 27.94	133.59 ± 40.26^*^	0.79 (0.67–0.91)	92.27 ± 24.00	114.23 ± 27.97^*^	1.25 (1.03–1.51)	123.74 ± 47.85	143.45 ± 29.33	1.21 (0.96–1.52)
AUC_inf_/D	105.41 ± 28.29	143.10 ± 43.67^*^	0.75 (0.64–0.87)	94.22 ± 24.54	116.58 ± 28.07^*^	1.25 (1.03–1.51)	132.87 ± 52.60	153.33 ± 30.98	1.21 (0.96–1.53)
t_1/2_ (h)	24.9 ± 5.8	35.3 ± 9.6^*^		23.3 ± 5.2	26.4 ± 6.2		34.3 ± 9.8	36.34 ± 1.05	

*C_max_/D, dose-normalized C_max_ value; T_max_, time to maximum plasma concentration; AUC_last_/D, dose-normalized AUC_last_ value; AUC_inf_/D, dose-normalized AUC_inf_ value; t_1/2_, terminal half-life. All values are presented as mean ± SD, except for T_max_, which is expressed as median (range). SD, standard deviation; GMR, geometric mean ratios; CI, confidence interval; CK, compound K*.

* *p-value < 0.05*.

#### Sex-related impacts on pharmacokinetic properties

No significant sex differences were observed in dose-normalized exposure parameters of the 100, 200, and 400 mg dose groups in the single-dose trial. In the multiple-dose trial, the PK parameters according to sexes on day 1 and day 15 are tabulated in Table 4. These parameters reveal that females had a higher dose-normalized C_max_ than males after administration of CK on day 1 and day 15 (14.49 ± 4.07 vs. 11.00 ± 4.25 ng·ml^−1^·mg^−1^, *p* = 0.025; 14.25 ± 3.38 vs. 11.18 ± 5.19 ng·ml^−1^·mg^−1^, *p* = 0.024), and significantly larger dose-normalized AUC_last_ and AUC_inf_ only on day 1 (114.23 ± 27.97 vs. 92.27 ± 24.00 h·ng·ml^−1^·mg^−1^, *p* = 0.025; 116.59 ± 28.07 vs. 94.22 ± 24.54 h·ng·ml^−1^·mg^−1^, *p* = 0.025), with the 90% CIs falling outside of the conventional 0.80–1.25. Although the AUC of CK in females was higher than that of males on day 15, the difference was not statistically significant. Both the T_max_ and t_1/2_ showed no significant sex dimorphism on either day 1 or day 15. The results of the two-way ANOVA revealed no interaction effects between repeated administration of CK and sex, with *F*_(1, 56)_ = 0.004, *p* = 0.950 for C_max_/D, *F*_(1, 56)_ = 0.042, *p* = 0.839 for AUC_last_/D, and *F*_(1, 44)_ = 0.047, *p* = 0.830 for AUC_inf_/D.

### Safety and tolerability

#### Vital signs, clinical laboratory tests, and ECG

There were significant differences in body temperature, heart rate, respiration, systolic and diastolic blood pressure at the different time points (*p* < 0.05). No statistically significant differences were detected in vital signs among the dose groups or between the test group and the placebo group, except for the body temperature, which varied among the different dose groups (*p* < 0.05). The above statistical differences were considered as not clinically significant based on the following reasons. First, body temperature, heart rate, respiration and blood pressure were within the normal range for all subjects and did not show a significant increase or decrease after drug administration. Second, these vital signs were greatly affected by environmental and psychological factors. Last, the indicators mentioned above were tested at single-point-of-time in two trials.

Additionally, the laboratory tests were mostly within the normal range or had no abnormality of clinical significance as judged by the doctor. Under the above conditions, some laboratory indexes, such as plasma triglyceride (TG), showed statistical differences (*p* < 0.05) between the test drug group and the placebo group in the single-dose trial, but these differences were not observed after multiple dosing. These differences might be resulted from the individual differences or normal physiological fluctuations and might have nothing to do with the test drug. Moreover, none of the subjects had clinically significant abnormalities in the ECG after the drug treatments.

#### Adverse events

In the single-dose trial, there were 44 cases of AEs that happened to 30 subjects (39.5%) among the 76 subjects enrolled in this trial. Of the 30 subjects who experienced AEs, 22 subjects suffered 33 AEs after taking the test drug while 8 subjects suffered 11 AEs after taking the placebo. The probability of AEs was 35.5% among the subjects who took Ginsenoside Compound K Tablets, and 57.1% among those who took placebo tables. Of the 44 cases of AEs, 13 cases were probably related to the test drug, while the other 7 cases were possibly related to CK. The most commonly reported drug-related AEs were diarrhea, abdominal pain, and direct bilirubin increased.

In the multiple-dose trial, there were 76 cases of AEs that happened to 31 subjects among the 36 subjects (86.1%) enrolled in this trial, with 10, 11, and 10 subjects in the 100, 200, and 400 mg groups experiencing 22, 26, and 28 cases of AEs, respectively. Of the 31 subjects who suffered adverse events, 69 cases of AEs arose among the 27 subjects (90.0%) who took CK tablets, while 7 cases of AEs arose in 4 subjects (70.0%) who took the placebo. Depending on the doctor's judgment, 20 cases of AEs were positively relevant to CK, with 23 cases being probably and 14 cases being possibly related to CK. Diarrhea occurred with the highest frequency and was regarded as a CK-related AE. We also observed that the CK exposure levels in subjects who suffered AEs in the gastrointestinal tract (diarrhea, abdominal pain) were significantly higher than those in subjects without gastrointestinal AEs.

In conclusion, the overall incidence of adverse reactions did not appear to be dose-dependent. The name and distribution of test drug related AEs (including positively, probably and possibly related AEs) in these trials are presented in Table 5.

**Table 5:** Summary of the number (%) of subjects experiencing study drug-related adverse events.

**AEs**	**Single-dose trial**							**Multiple-dose trial**		
	**25 mg (*n* = 8)**	**50 mg (*n* = 8)**	**100 mg (*n* = 10)**	**200 mg (*n* = 10)**	**400 mg (*n* = 10)**	**600 mg (*n* = 8)**	**800 mg (*n* = 8)**	**100 mg (*n* = 10)**	**200 mg (*n* = 10)**	**400 mg (*n* = 10)**
**Any AEs**	0	2 (2.6)	2 (2.6)	5 (6.6)	5 (6.6)	1 (1.3)	3 (3.9)	7 (19.4)	10 (27.8)	9 (25)
**GASTROINTESTINAL**										
Diarrhea	0	0	0	2 (2.6)	2 (2.6)	0	1 (1.3)	3 (8.3)	9 (25)	9 (25)
Bellyache	0	0	0	0	1 (1.3)	0	1 (1.3)	0	4 (11.1)	1 (2.8)
**CLINICAL LABORATORY STUDIES**										
Direct bilirubin increased	0	2 (2.6)	0	2 (2.6)	1 (1.3)	1 (1.3)	0	2 (5.6)	1 (2.8)	5 (13.9)
Total bilirubin increased	0	0	0	1 (1.3)	0	0	0	0	1 (2.8)	3 (8.3)
Alkaline phosphatase increased	0	0	0	1 (1.3)	0	0	0	0	1 (2.8)	0
Blood white cells increased	0	0	0	0	1 (1.3)	0	0	0	0	0
**OTHER AEs**										
Rash	0	0	0	0	0	0	1 (1.3)	0	0	0
Subcutaneous bleeding (double forearm)	0	0	0	0	0	0	0	0	0	1 (2.8)
Headache	0	0	1 (1.3)	0	1 (1.3)	0	0	0	0	0
Dizziness	0	0	1 (1.3)	0	0	0	0	4 (11.1)	1 (2.8)	0
Nausea	0	0	0	0	0	0	0	1 (2.8)	0	0
Loss of appetite	0	0	0	0	0	0	0	2 (5.6)	0	0

*All values are expressed as number of subjects (%). AEs, adverse events*.

## Discussion

This study was designed to evaluate the pharmacokinetics, safety and tolerability of the ginsenoside CK after a range of single and multiple oral doses of CK in healthy Chinese volunteers. Additionally, we also investigated the impact of sex on the response to the exposure to CK. PK analysis such as the current study was necessary for the determination of the appropriate clinical dose and the potential dose adjustment in key subpopulations.

PK studies of ginsenoside CK have been particularly limited, and the existing clinical trials about CK were conducted using ginseng extract and not the pure compound (Lee et al., 2009; Jin et al., 2012; Kim, 2013; Kim et al., 2013). Compared with this study, the exposure of CK was at a far lower level, albeit oral dose was much higher, as CK is absent in nature and needs to be biotransformed from natural/fermented extracts. In addition, not surprisingly, the T_max_ was significantly prolonged (T_max_ of 2.5–3.8 h in this study) because biotransformation takes time. Simultaneously, almost without exception, the trials suggested that the CK pharmacokinetics differed significantly among individuals. However, it was difficult to tell whether this difference was due to intestinal transformation activity or the properties of the CK itself. The PK parameters, such as C_max_ and the AUC, displayed large variations resulting in SD values that were even larger than half the mean value in this study, indicating that the significant individual difference was at least partly caused by the characteristics of CK. All things considered, it was not difficult to infer that the action mechanism of CK *in vivo* is complicated and may be mediated through a wide variety of phase I and II metabolizing enzymes and/or transporters. To date, there have been several reports about the interaction between CK and P-glycoprotein (P-gp), a classic intestinal efflux protein (Yang et al., 2012; Zhang et al., 2012; Li et al., 2014), but it is not yet clear whether CK is a substrate or an inhibitor of P-gp. Pharmacokinetics analysis of CK in rats and on Caco-2 cell monolayers conducted by Paek et al. suggested that the CK might be mediated through the hepatic uptake transporters (Paek et al., 2006). Additionally, there was not any report on the relationship between CK and other transporters. As for metabolizing enzyme, Xiao et al. found that CK was a substrate and also a moderate inhibitor for both CYP2C9 and CYP3A4 through *in vitro* experiment performed on human liver microsomes (HLMs) using human recombinant CYPs (Xiao et al., 2016). Accordingly, the high inter- and intra-variability in the expressions or activities of metabolizing enzymes and transporters may contribute to the individual differences of CK. We believe that the exclusion of three subjects in single-dose trial for serious deflective PK parameters was also responsible for this reason. Since the limited number of subjects in phase I clinical trials, it is likely that only a few mutants were included. Nevertheless, the existing evidence still cannot fully explain the complicated mechanism of action of CK *in vivo*. Related studies have been undertaken in our laboratory to clarify the ADME process of CK.

The increases in C_max_ and AUC of CK were dose-proportional over the dosage range 100–400 mg/day, either after a single dose or repeated administrations qd for 9 consecutive days. In addition, the PK parameter AUC_τ_, which reflects the exposure to the drug during the long-term medication of multiple doses, showed a linear increase with the dose increase. However, the nonlinear increase of the exposure with dose (25–800 mg) displayed a nonlinear pharmacokinetics of CK. As previously discussed, CK might be mediated by transporters *in vivo*, thus we put forward the following hypothesis. The nonlinear pharmacokinetics exhibited outside the range of 100–400 mg of CK could be due to saturated secretion mediated by apical membrane transporters (e.g., P-gp) at low doses and the limitation of drug dissolution in the intestine at high doses of CK. Within the range of 100–400 mg of CK, we assume that the dissolution of CK in the intestine is proportional to the dose, and the efflux transporters are concurrently saturated. However, this is still a hypothesis that we have put forward, and needs to be intensively investigated in the future. The accumulation of CK was considered to be moderate, with an accumulation index that was approximately equal to 2.66 (Li et al., 2013). Similarly, t_1/2_ was much longer on day 15, which also indicated that CK would accumulate *in vivo* after repeated administrations.

Our previous study results revealed that CK exposure in females was significantly higher than in males after oral administration (Supplementary Figures 1, 2 and Supplementary Table 1), and pharmacokinetics data in rats suggested that the sex dimorphism mainly happened in the absorption phase of CK. Another clinical trial (registration number ChiCTR-IPR-15005787), designed to evaluate the effects of food and sex on the pharmacokinetics of CK, confirmed that its PK property was sex-related, and this dimorphism was independent from the food effect (Chen et al., 2017). We therefore hypothesized that intestinal transporters play a major role in CK mechanism of action *in vivo* for the following reasons. On the one hand, the expression and activity of certain transporters may have primeval sex differences. On the other hand, potential interactions between CK and certain transporters may exist so that CK could promote the emergence of sex differences. Indeed, some studies have reported the sex-related expression of transporters in target organs (Rost et al., 2005; Tornatore et al., 2013; Pastor et al., 2016). Unfortunately, due to the difficulties in distinguishing intestinal segments and inconsistency among protein levels, mRNA levels, and activities, the sex-related expression of transporters in the intestine still remains controversial (MacLean et al., 2008; Mariana et al., 2011). Another phenomenon that we could observe from these two trials was that food can significantly increase the absorption of CK, which was consistent with the results from another independent clinical trial (Chen et al., 2017). The exact mechanism for the increased ginsenoside CK exposure in the presence of a high-fat meal is still unknown and may be the result of increasing solubilization and dissolution in the intestinal fluids, especially, for the drugs with poor water solubility (Welling, 1996). In addition, drug-food interactions (DFI) may change systemic exposure of the drug by blocking or activating certain transporters in the intestine (Nakanishi and Tamai, 2015). In summary, the phenomena of sex differences and DFI for CK may be caused by a single transporter e.g., P-gp or more. Furthermore, the sex differences in the pharmacokinetics of CK may also be due to the intricate interrelations of the drug, food, hormone and intestinal flora. Further studies are currently being conducted in our laboratory to elucidate the interactions between intestinal transporters and CK.

After a single dose of CK ranging from 25 to 800 mg, and multiple doses of 100–400 mg, CK was found to be safe and well-tolerated in healthy Chinese volunteers, with no significant safety concerns and no serious AEs reported in these two trials. The most commonly observed and drug-related AEs were diarrhea and abdominal pain, and the frequency was exposure-related after we analyzed. Since we also observed this phenomenon occurring in animals, we considered diarrhea and abdominal pain as being AEs positively related to CK. Drug-induced diarrhea was found to be a common adverse drug reaction accounting for about 7% of all adverse drug reactions (Abraham and Sellin, 2007). In another report, 5 of the 24 subjects experienced diarrhea after taking the fermented ginseng extract containing CK (Jin et al., 2012). The mechanism of the diarrheal effect of CK is being studied in order to provide a theoretical basis and guidance for the approval of the application of CK or development of a better CK formulations with less adverse reactions in the future. Moreover, according to the doctor's judgment and the statistical analysis of the laboratory tests results, the most frequent and may be drug related laboratory abnormality was elevated direct bilirubin. Although it occurred in both the test drug and placebo groups, a relationship between CK and AEs cannot be excluded. In conclusion, all observed AEs were mild or moderate, and the vast majority of them were reversible and disappeared without any treatment. Based on the above data, the moderate CK accumulation after repeated administrations should not cause any lasting, serious harm to human. Future multi-center trials including a larger sample of RA patients will be carried out to verify the AEs related to CK. According to this study, the safety results observed were promising.

This work and what we've already published (Chen et al., 2017) both belong to phase I clinical trials of Ginsenoside Compound K Tablets (Hisun Pharmaceutical Co., Ltd, Taizhou, China), but they are completely independent trials. More concretely, these two studies were performed on different subjects through different designs for different purposes. This dose-ascending study was to investigate the PK properties, safety and tolerability of CK, which help to determine the dose in clinical application. While the published one (Chen et al., 2017) was to evaluate the effects of food and sex on CK and PPD, which provides the basis for clinical medication and individual drug administration. Either of them is a key step of the research that goes into developing a new drug. As preliminary clinical researches, these trials greatly enriched the PK knowledge of CK and provide particularly useful research directions for this promising drug candidate.

Undoubtedly, more experiments need to be carried out to corroborate the sex dimorphism observed in this study and our hypotheses about the mechanism behind this phenomenon. Although there is still a long way to go to guide the clinical application of CK from this study, at least these trials suggest that we must take repeated administration, sex and food factors into account in future studies. Currently, phase II clinical trials of Ginsenoside Compound K Tablets are due to begin, as it is a drug product for investigational new drugs (INDs) applications. In addition, a series of *in vitro* and *in vivo* studies have been conducted, which may contribute to explain the characteristics of the pharmacokinetics of CK.

## Conclusions

In summary, CK was safe and well-tolerated in healthy Chinese volunteers and displayed a linear increase in the C_max_ and AUC values at single-daily dose ranging from 100 to 400 mg. A moderate accumulation occurred after multiple administrations. Food was a definite factor that can significantly promote the absorption of CK, and the impact of sex on the pharmacokinetics of CK still requires further investigation.

## Author contributions

LC and LZ: contributed equally to the study design, process of samples, data analysis, and article writing; JH and YQW: assisted with the study design and data analysis; GY, JH, and JL: were involved in the implementation of trials; ZT and YCW: participated in chromatographic analysis; DO: was the principal investigator of this trial and was involved in the study design and data collection. All authors earnestly reviewed and approved the final version for this manuscript, and assume overall responsibility for the accuracy of data analysis and reporting.

### Conflict of interest statement

The authors declare that the research was conducted in the absence of any commercial or financial relationships that could be construed as a potential conflict of interest.

## Abbreviations

G-CK: ginsenosides compound K
CK: compound K
NSAIDs: nonsteroidal anti-inflammatory drugs
DMARDs: disease modifying antirheumatic drugs
PPD: protopanaxadiol
PK: pharmacokinetic
ADME: absorption, distribution, metabolism and excretion
HED: human equivalent dose
MRSD: maximum recommended starting dose
IS: internal standard
CVs: coefficient of variations
DFI: drug-food interactions.

## References

[B1] Abraham B. Sellin J. H. (2007). Drug-induced diarrhea. Curr. Gastroenterol. Rep. 9, 365–372. 10.1007/s11894-007-0044-x 17991336

[B2] Bren L. (2005). Does sex make a difference? FDA Consum. 39, 10–15. Available online at: https://permanent.access.gpo.gov/lps1609/www.fda.gov/fdac/features/2005/405_sex.html 16252395

[B3] Chen J. Wu H. Wang Q. Chang Y. Liu K. Wei W. (2015). Ginsenoside metabolite compound K suppresses T-cell priming via modulation of dendritic cell trafficking and costimulatory signals, resulting in alleviation of collagen-induced arthritis. J. Pharmacol. Exp. Ther. 353, 71–79. 10.1124/jpet.114.220665 25630466

[B4] Chen L. Zhou L. Wang Y. Yang G. Huang J. Tan Z.. (2017). Food and sex-related impacts on the pharmacokinetics of a single-dose of ginsenoside compound K in healthy subjects. Front. Pharmacol. 8:636. 10.3389/fphar.2017.00636 28955238 PMC5602130

[B5] Clayton J. A. Collins F. S. (2014). Policy: NIH to balance sex in cell and animal studies. Nature 509, 282–283. 10.1038/509282a 24834516 PMC5101948

[B6] Couzin-Frankel J. (2014). National Institutes of Health. Needed: more females in animal and cell studies. Science 344:679. 10.1126/science.344.6185.679 24833367

[B7] Cuong T. T. Yang C. S. Yuk J. M. Lee H. M. Ko S. R. Cho B. G.. (2009). Glucocorticoid receptor agonist compound K regulates Dectin-1-dependent inflammatory signaling through inhibition of reactive oxygen species. Life Sci. 85, 625–633. 10.1016/j.lfs.2009.08.014 19733186

[B8] Institute of Medicine (US) Board on Population Health and Public Health Practice (2012). Sex-Specific Reporting of Scientific Research: A Workshop Summary. Washington, DC: National Academies Press (US). 22379657

[B9] Institute of Medicine (US) Forum on Neuroscience and Nervous System Disorders (2011). Sex Differences and Implications for Translational Neuroscience Research: Workshop Summary. Washington, DC: National Academies Press (US). 21452459

[B10] Jin H. Seo J. H. Uhm Y. K. Jung C. Y. Lee S. K. Yim S. V. (2012). Pharmacokinetic comparison of ginsenoside metabolite IH-901 from fermented and non-fermented ginseng in healthy Korean volunteers. J. Ethnopharmacol. 139, 664–667. 10.1016/j.jep.2011.11.052 22178175

[B11] Joh E. H. Lee I. A. Jung I. H. Kim D. H. (2011). Ginsenoside Rb1 and its metabolite compound K inhibit IRAK-1 activation–the key step of inflammation. Biochem. Pharmacol. 82, 278–286. 10.1016/j.bcp.2011.05.003 21600888

[B12] Kim H. K. (2013). Pharmacokinetics of ginsenoside Rb1 and its metabolite compound K after oral administration of Korean Red Ginseng extract. J. Ginseng Res. 37, 451–456. 10.5142/jgr.2013.37.451 24235859 PMC3825860

[B13] Kim J. S. Kim Y. Han S. H. Jeon J. Y. Hwang M. Im Y. J.. (2013). Development and validation of an LC-MS/MS method for determination of compound K in human plasma and clinical application. J. Ginseng Res. 37, 135–141. 10.5142/jgr.2013.37.135 23717167 PMC3659617

[B14] Lee J. Lee E. Kim D. Lee J. Yoo J. Koh B. (2009). Studies on absorption, distribution and metabolism of ginseng in humans after oral administration. J. Ethnopharmacol. 122, 143–148. 10.1016/j.jep.2008.12.012 19146939

[B15] Li L. Li X. Xu L. Sheng Y. Huang J. Zheng Q. (2013). Systematic evaluation of dose accumulation studies in clinical pharmacokinetics. Curr. Drug Metab. 14, 605–615. 10.2174/13892002113149990002 23701162

[B16] Li N. Wang D. Ge G. Wang X. Liu Y. Yang L. (2014). Ginsenoside metabolites inhibit P-glycoprotein *in vitro* and in situ using three absorption models. Planta Med. 80, 290–296. 10.1055/s-0033-1360334 24493631

[B17] MacLean C. Moenning U. Reichel A. Fricker G. (2008). Closing the gaps: a full scan of the intestinal expression of p-glycoprotein, breast cancer resistance protein, and multidrug resistance-associated protein 2 in male and female rats. Drug Metab. Dispos. 36, 1249–1254. 10.1124/dmd.108.020859 18378562

[B18] Mariana B. Adrián L. Guillermo V. Juan S. Laura M. Carlos L. (2011). Gender-related differences on P-glycoprotein-mediated drug intestinal transport in rats. J. Pharm. Pharmacol. 63, 619–626. 10.1111/j.2042-7158.2010.01230.x 21492163

[B19] Nakanishi T. Tamai I. (2015). Interaction of drug or food with drug transporters in intestine and liver. Curr. Drug Metab. 16, 753–764. 10.2174/138920021609151201113537 26630906

[B20] NIH (2001). NIH Policy and Guideline on the Inclusion of Women and Minorities as Subjects in Clinical Research Notice NOT-OD-02-001. Bethesda, MD: National Institutes of Health.

[B21] Oh J. Kim J. S. (2016). Compound K derived from ginseng: neuroprotection and cognitive improvement. Food Funct. 7, 4506–4515. 10.1039/C6FO01077F 27801453

[B22] Paek I. B. Moon Y. Kim J. Ji H. Y. Kim S. A. Sohn D. H.. (2006). Pharmacokinetics of a ginseng saponin metabolite compound K in rats. Biopharm. Drug Dispos. 27, 39–45. 10.1002/bdd.481 16302287

[B23] Park E. S. Lee K. P. Jung S. H. Lee D. Y. Won K. J. Yun Y. P.. (2013). Compound K, an intestinal metabolite of ginsenosides, inhibits PDGF-BB-induced VSMC proliferation and migration through G1 arrest and attenuates neointimal hyperplasia after arterial injury. Atherosclerosis 228, 53–60. 10.1016/j.atherosclerosis.2013.02.002 23473423

[B24] Pastor L. Vettorazzi A. Campión J. Cordero P. López de Cerain A. (2016). Gene expression kinetics of renal transporters induced by ochratoxin A in male and female F344 rats. Food Chem. Toxicol. 98, 169–178. 10.1016/j.fct.2016.10.019 27771458

[B25] Rost D. Kopplow K. Gehrke S. Mueller S. Friess H. Ittrich C.. (2005). Gender-specific expression of liver organic anion transporters in rat. Eur. J. Clin. Invest. 35, 635–643. 10.1111/j.1365-2362.2005.01556.x 16178883

[B26] USA Report to Congressional Requesters. (2000). NIH has Increased its Efforts to Include Women in Research (GAO/HEH-00-96). Report to Congressional Requesters. Washington, DC: United States General Accounting Office.

[B27] Tawab M. A. Bahr U. Karas M. Wurglics M. Schubert-Zsilavecz M. (2003). Degradation of ginsenosides in humans after oral administration. Drug Metab. Dispos. 31, 1065–1071. 10.1124/dmd.31.8.1065 12867496

[B28] Tornatore K. M. Brazeau D. Dole K. Danison R. Wilding G. Leca N.. (2013). Sex differences in cyclosporine pharmacokinetics and ABCB1 gene expression in mononuclear blood cells in African American and Caucasian renal transplant recipients. J. Clin. Pharmacol. 53, 1039–1047. 10.1002/jcph.123 23908147

[B29] Wakabayashi C. Hasegawa H. Murata J. Saiki I. (1997). *In vivo* antimetastatic action of ginseng protopanaxadiol saponins is based on their intestinal bacterial metabolites after oral administration. Oncol. Res. 9, 411–417. 9436194

[B30] Wang C. Z. Du G. J. Zhang Z. Wen X. D. Calway T. Zhen Z.. (2012). Ginsenoside compound K, not Rb1, possesses potential chemopreventive activities in human colorectal cancer. Int. J. Oncol. 40, 1970–1976. 10.3892/ijo.2012.1399 22426808 PMC3349346

[B31] Wang Y. Chen J. Luo X. Zhang Y. Si M. Wu H.. (2016). Ginsenoside metabolite compound K exerts joint-protective effect by interfering with synoviocyte function mediated by TNF-α and Tumor necrosis factor receptor type 2. Eur. J. Pharmacol. 771, 48–55. 10.1016/j.ejphar.2015.12.019 26688568

[B32] Welling P. G. (1996). Effects of food on drug absorption. Annu. Rev. Nutr. 16, 383–415. 10.1146/annurev.nu.16.070196.002123 8839932

[B33] Wu H. Chen J. Wang Q. Jia X. Song S. Yuan P.. (2014). Ginsenoside metabolite compound K attenuates inflammatory responses of adjuvant-induced arthritis rats. Immunopharmacol. Immunotoxicol. 36, 124–129. 10.3109/08923973.2014.880717 24450920

[B34] Xiao J. Chen D. Lin X. X. Peng S. F. Xiao M. F. Huang W. H.. (2016). Screening of drug metabolizing enzymes for the ginsenoside compound K *in vitro*: an efficient anti-cancer substance originating from Panax Ginseng. PLoS ONE 11:e0147183. 10.1371/journal.pone.0147183 26845774 PMC4742234

[B35] Yan Q. Zhou X. W. Zhou W. Li X. W. Feng M. Q. Zhou P. (2008). Purification and properties of a novel beta-glucosidase, hydrolyzing ginsenoside Rb1 to CK, from Paecilomyces Bainier. J. Microbiol. Biotechnol. 18, 1081–1089. 10.1271/bbb.70425 18600051

[B36] Yang X. D. Yang Y. Y. Ouyang D. S. Yang G. P. (2015). A review of biotransformation and pharmacology of ginsenoside compound K. Fitoterapia 100, 208–220. 10.1016/j.fitote.2014.11.019 25449425

[B37] Yang Z. Wang J. R. Niu T. Gao S. Yin T. You M.. (2012). Inhibition of P-glycoprotein leads to improved oral bioavailability of compound K, an anticancer metabolite of red ginseng extract produced by gut microflora. Drug Metab. Dispos. 40, 1538–1544. 10.1124/dmd.111.044008 22584255 PMC3400789

[B38] Yoo M. H. Yeom S. J. Park C. S. Lee K. W. Oh D. K. (2011). Production of aglycon protopanaxadiol via compound K by a thermostable beta-glycosidase from *Pyrococcus furiosus*. Appl. Microbiol. Biotechnol. 89, 1019–1028. 10.1007/s00253-010-2960-1 21052989

[B39] Yosioka I. Sugawara T. Imai K. Kitagawa I. (1972). Soil bacterial hydrolysis leading to genuine aglycone. V. On ginsenosides-Rb1, Rb2, and Rc of the ginseng root saponins. Chem. Pharm. Bull. 20, 2418–2421. 10.1248/cpb.20.2418

[B40] Zhang B. Zhu X. M. Hu J. N. Ye H. Luo T. Liu X. R.. (2012). Absorption mechanism of ginsenoside compound K and its butyl and octyl ester prodrugs in Caco-2 cells. J. Agric. Food Chem. 60, 10278–10284. 10.1021/jf303160y 23013417

