# Supplementary material for: Single- and Multiple-Dose Trials to Determine the Pharmacokinetics, Safety, Tolerability, and Sex Effect of Oral Ginsenoside Compound K in Healthy Chinese Volunteers
Source: Front Pharmacol. 2018 Jan 11;8:965. doi: 10.3389/fphar.2017.00965 (PMC5769417; doi:10.3389/fphar.2017.00965)
Supplement: Supplementary file 1 [file Table1.DOC]

Supplementary Material

**Single- and multiple-dose trials to determine the pharmacokinetics, safety, tolerability, and sex effect of oral Ginsenoside compound K in healthy Chinese volunteers**

Lulu Chen^1,2^**^*^**, Luping Zhou^1,2^**^*^**, , Jie Huang^3^, Yaqin Wang^1,2^, Guoping Yang^3^, Zhirong Tan^1,2^, Yicheng Wang^1,2^, Gan Zhou^1,2^,, Jianwei Liao^1,2^, Dongsheng Ouyang^1,2^

***These authors contributed equally to this work.**

*** Correspondence:** Dongsheng Ouyang: [ouyangyj@163.com](mailto:ouyangyj@163.com)

**Supplemental Table 1.** Sex differences of ginsenoside compound K in human

| **Parameter** | **Male (n = 12)** | **Female (n = 12)** |
| --- | --- | --- |
| C_max_/D | 16.44 ± 2.82 | 24.87 ± 8.23* |
| T_max_ | 3.3 (2.0-6.0) | 2.8 (2.0-4.0) |
| t_1/2_ | 6.1 ± 1.1 | 6.7 ± 1.2 |
| AUC_last_/D | 387.86 ± 174.14 | 1984.33 ± 1809.10* |
| AUC_inf_/D | 1236.97 ± 6.8.25 | 2016.33 ± 1124.90* |

C_max_/D, dose-normalized C_max_ value; T_max_, time to maximum plasma concentration; t_1/2_, terminal half-life; AUC_last_/D, dose-normalized AUC_last_ value; AUC_inf_/D, dose-normalized AUC_inf_ value; All values are presented as mean ± SD, except for T_max_, which is expressed as median (range). SD, standard deviation. PK parameters were compared between male and female by independent-sample t-test. A non-parametric test was performed on the T_max_ and t_1/2_. * Compared with Male, *p* < 0.05. These were the results of phase 0 trial, which was a cross-over clinical trial and included 12 males and 12 females.
