# Supplementary material for: Single- and Multiple-Dose Trials to Determine the Pharmacokinetics, Safety, Tolerability, and Sex Effect of Oral Ginsenoside Compound K in Healthy Chinese Volunteers
Source: Front Pharmacol. 2018 Jan 11;8:965. doi: 10.3389/fphar.2017.00965 (PMC5769417; doi:10.3389/fphar.2017.00965)
Supplement: Supplementary file 2 [file Image1.PDF]

frontiers

Supplementary Material

Single- and multiple-dose trials to determine the pharmacokinetics, safety, tolerability, and sex effect of oral Ginsenoside compound K in healthy Chinese volunteers

Lulu Chen[1,2*], Luping Zhou[1,2*], , Jie Huang[3], Yaqin Wang[1,2], Guoping Yang[3], Zhirong Tan[1,2], Yicheng Wang[1,2], Gan Zhou[1,2,], Jianwei Liao[1,2], Dongsheng Ouyang[1,2]

***These authors contributed equally to this work.**

*** Correspondence:** Dongsheng Ouyang: ouyangyj@163.com

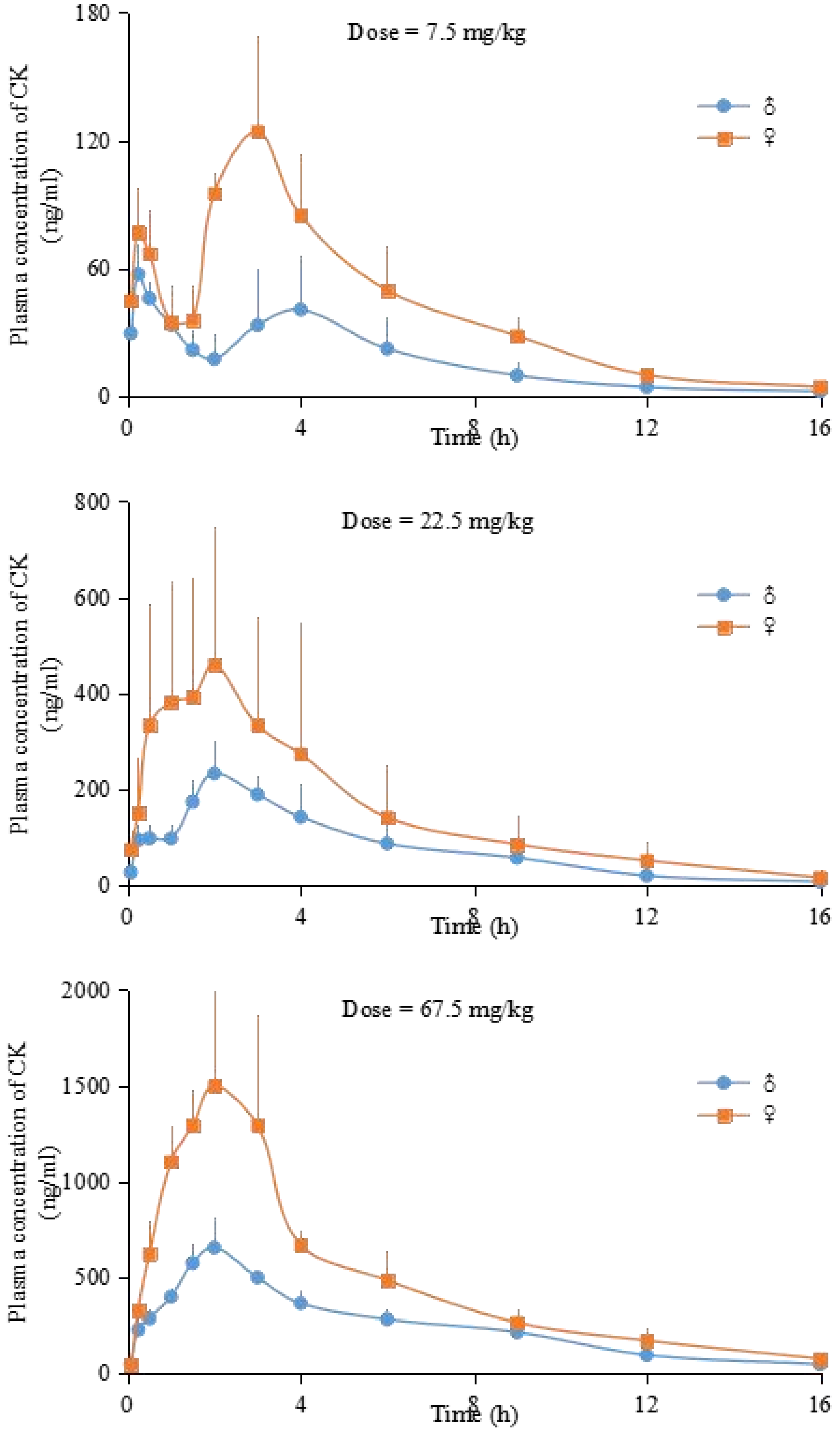

Supplementary Figure 1. Pharmacokinetic profiles (Mean ± SD) of CK in rats after intragastric administration of CK. SD, standard deviation; CK, compound K.
